# Worldwide Dissemination of the *bla*_OXA-23_ Carbapenemase Gene of *Acinetobacter baumannii*^1^

**DOI:** 10.3201/eid1601.090852

**Published:** 2010-01

**Authors:** Pauline D. Mugnier, Laurent Poirel, Thierry Naas, Patrice Nordmann

**Affiliations:** Institut National de la Santé et de la Recherche Médicale Unité 914, Paris, France; Université Paris Sud, Bicêtre, France; 1 This work was presented in part at the 19th European Congress of Clinical Microbiology and Infectious Diseases, Helsinki, Finland, May 16–19, 2009.

**Keywords:** Acinetobacter baumannii, oxacillinase, carbapenems, Tn2006, ISAba1, bacteria, zoonoses, infectious diseases, vector-borne infections, research

## Abstract

To assess dissemination of OXA-23–producing strains of *Acinetobacter baumannii*, we obtained 20 carbapenem-resistant, OXA-23–producing isolates from different regions. Their clonal relationship was assessed by pulsed-field gel electrophoresis and multilocus sequence typing. We identified 8 sequence types, including 4 novel types. All except 2 strains belonged to 2 main European clonal lineages. The *bla*_OXA-23_ gene was either located on the chromosome or on plasmids and associated with 4 genetic structures.

## Abstract 2

Controlling the spread of this gene will be difficult.

*Acinetobacter baumannii* is a gram-negative organism that is increasingly recognized as a major pathogen causing nosocomial infections, including bacteremia and ventilator-associated pneumonia, particularly in patients admitted to intensive care units (*1*). Several studies have shown the geographically widespread occurrence of multidrug-resistant *A*. *baumannii* strains, which suggested a clonal relatedness of these strains. Three international *A*. *baumannii* clones associated with multidrug resistance (European clones I, II, and III) have been reported (*2*).

Increasing resistance to carbapenems has been observed worldwide in the past decade, frequently mediated by production of class D β-lactamases with carbapenemase activity. Three acquired class D β-lactamases with carbapenemase gene clusters have been described in *A*. *baumannii*, which correspond to *bla*_OXA-23_-like, *bla*_OXA-40_-like, and *bla*_OXA-58_-like genes (*3*). The *bla*_OXA-23_ gene, first characterized in Scotland (*4*), has been increasingly reported worldwide. *A*. *radioresistens* was recently identified as the progenitor of the *bla*_OXA-23_-like genes (*5*). Clonal outbreaks of carbapenem-resistant and OXA-23–producing *A*. *baumannii* have been reported in many countries, such as Bulgaria (*6*), People’s Republic of China (*7*), Brazil (*8*), Iraq (*9*), Afghanistan (*9*), and French Polynesia (*10*).

Genetic acquisition of the *bla*_OXA-23_ gene was investigated and transposons Tn*2006*, Tn*2007*, and Tn*2008* were identified as genetic structures harboring this gene (*10**–**12*). In Tn*2006*, the *bla*_OXA-23_ gene is flanked by 2 copies of the insertion sequence IS*Aba1*, which are located in opposite orientations (Figure 1). The functionality of Tn*2006* has been recently demonstrated (*13*). Tn*2008* is similar to Tn*2006* but lacks the second copy of IS*Aba1* and the *bla*_OXA-23_ gene is associated with 1 copy of IS*Aba4* (which differs from IS*Aba1*) in Tn*2007* (Figure 1) (*11*). As reported for strains from United Arab Emirates and Bahrain, the *bla*_OXA-23_ gene can be associated with only 1 copy of IS*Aba1* (*14**,**15*). We studied the clonal relationship and genomic environment of sequences surrounding the *bla*_OXA-23_ gene among a collection of OXA-23–producing isolates from 15 countries.

**Figure 1:**
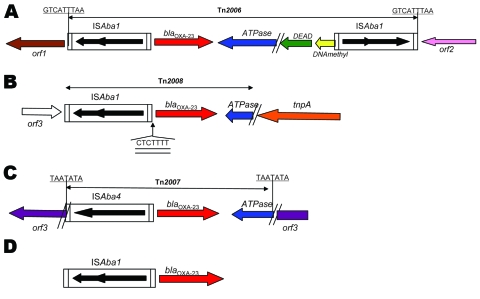
Genetic structures associated with the *bla*_OXA-23_ gene of *Acinetobacter baumannii*. A) Tn*2006* from isolates 240, 512, 810, 859, 883 and AUS (ST22/ST2). B) Tn*2008* from isolate 614. C) Tn*2007* from isolates Ab14, BEL, and DOS. D) IS*Aba1* from isolates AS3, 1190, 861, and 877. Boundaries of Tn*2006*, Tn*2007*, and Tn*2008* are indicated with the target site duplication likely generated by transposition events underlined. The 7-bp difference in the site of insertion of IS*Aba1* for isolate 614 is double-underlined. The open reading frame 1 (*orf1*), *orf2*, and *orf3* genes of unknown function is indicated. *tnpA*, gene encoding a putative transposase; *ATPase*, gene encoding the putative AAA ATPase; *DEAD*, gene encoding the putative DEAD (Asp-Glu-Ala-Asp) helicase; DNAmethyl, DNA methylase.

## Materials and Methods

### Bacterial Strains and Susceptibility Testing

Twenty OXA-23–producing *A*. *baumannii* clinical isolates were obtained from 15 countries. These isolates had been obtained from patients hospitalized in intensive care units from December 2003 through March 2008. Isolates were obtained from tracheal aspirates (n = 3), bile (n = 1), urine (n = 4), wounds (n = 1), respiratory tract (n = 1), blood (n = 4), and sputum (n = 1). The isolates were initially chosen after preliminary pulsed-field gel electrophoresis (PFGE)–based typing had identified 13 pulsotypes. Isolates were obtained from France (n = 4), Vietnam (n = 1), New Caledonia (n = 1), Thailand (n = 1), Australia (n = 1), Tahiti (n = 1), Reunion (n = 2), South Africa (n = 1), United Arab Emirates (n = 2), Libya, (n = 1), Bahrain (n = 1), Egypt (n = 1), Belgium (n = 1), Algeria (n = 1), and Brazil (n = 1).

Presence of the *bla*_OXA-23_ gene was screened by PCR by using specific primers (OXA-23-A 5′-GGAATTCCATGAATAAATATTTTACTTGC-3′ and OXA-23-B 5′-CGGGATCCCGTTAAATAATATTCAGGTC-3′) and additional sequencing (ABI 3100 sequencer; Applied Biosystems, Foster City, CA, USA). Susceptibility patterns to β-lactam antimicrobial drugs were determined by using a standard disk diffusion method according to published standards (*16*) and Etest strips (AB Biodisk, Solna, Sweden). Isolates were identified by using 16S rRNA gene sequencing (*17*).

### Clonal Relationships

Isolates were typed by using *Apa*I macrorestriction analysis and PFGE according to the manufacturer’s recommendations (Bio-Rad, Marnes-la-Coquette, France). Bacteria were grown in a medium appropriate for the strain until an optical density of 0.8 to 1 at 600 nm was reached. One milliliter of cells was centrifuged, washed, and resuspended in 10 mmol/L Tris, pH 7.2, 20 mmol/L NaCl, 50 mmol/L EDTA. Immediately after resuspension, an equal volume of 2% low melting point InCert agarose (Bio-Rad) was added. Solid agarose plugs were lysed at 37°C for 2 h in 1 mL of lysis buffer (10 mmol/L Tris, pH 7.2, 50 mmol/L NaCl, 0.5% sodium laurylsarcosine, 0.2% sodium deoxycholate) supplemented with 20 mg/L of lysozyme. The plugs were then incubated at 55°C for 16 h with proteinase K buffer (100 mmol/L EDTA, pH 8, 0.2% sodium deoxycholate, 1% sodium laurylsarcosine) supplemented with 20 mg/L of proteinase K. Plugs were washed with Tris-EDTA buffer containing 1 mmol/L phenylmethylsulfonyl fluoride (Sigma, St. Louis, MO, USA) and 3× with Tris-EDTA buffer at room temperature.

Whole-cell DNA of *A*. *baumannii* isolates was digested with *Apa*I overnight at room temperature (New England Biolabs, St. Quentin-en-Yvelines, France). Electrophoresis was performed on a 1% agarose gel with 0.5× Tris-borate-EDTA buffer by using a CHEF DRII apparatus (Bio-Rad). Samples were subjected to electrophoresis at 14°C, 6 volts/cm, and a switch angle with 1 linear switch ramp of 3–8 s for 10.5 h, and then for 12–20 s for 10.5 h.

Identification of PCR-based sequence groups was conducted by using 2 multiplex PCR assays designed to selectively amplify group 1 or group 2 alleles of the gene encoding outer-membrane protein A (*ompA*), the gene encoding part of a pilus assembly system required for biofilm formation (*csuE*), and the gene encoding the intrinsic carbapenemase gene of *A*. *baumannii*) (*bla*_OXA-51_) (*18*). Clonal relationships were established by multilocus sequence typing (MLST) by using 7 standard housekeeping loci (citrate synthase [*gltA*], gyrase B [*gyrB*], glucose dehydrogenase B [*gdhB*], recombination A [*recA*], chaperone 60 [*cpn60*], glucose-6-phosphate isomerase [*gpi*], and RNA polymerase [*rpoD*]) as described (*18*). Sequencing of internal fragments was performed by using BigDye fluorescent terminators and primers described (*19*). Sequences were compared with the *A*. *baumannii* database at the MLST Website (http://mlst.zoo.ox.ac.uk). To supplement epidemiologic results, we performed a second MLST typing using the scheme developed by Nemec et al. (*20*). Sequences of the 7 housekeeping genes were analyzed by using an *A*. *baumannii* database (www.pasteur.fr/recherche/genopole/PF8/mlst/Abaumannii.html).

### Southern Blot Analysis and Location of *bla*_OXA-23_ Gene

Southern blot analysis was performed by using total genomic DNA digested with *Eco*RI, separated by electrophoresis on 0.8% agarose gels, transferred onto Hybond N+ membranes, and hybridized with enhanced chemiluminescence labeled probes overnight at 42°C. The membranes were developed according to the manufacturer’s instructions (GE Healthcare, Saclay, France). Chromosomal or plasmid locations of the β-lactamase gene were assessed by hybridization of I-*Ceu*I–digested genomic DNA with *bla*_OXA-23_ and 16S rDNA probes and electrophoresis (20–120 s for 9 h and 60–100 s for 11 h at 14°C and 5 V/cm^2^) (*21*). DNA was transferred from an agarose gel to a nylon membrane by capillary transfer. Hybridization, labeling, and detection were conducted as described above. Mating-out assays were performed by using isolates that had plasmid-borne *bla*_OXA-23_ as donors and rifampin-resistant *A*. *baumannii* BM4547 as recipients as described (*22*). Transconjugants were selected on trypticase soy agar plates containing ticarcillin (50 mg/L) and rifampin (50 mg/L).

### Cloning Experiments

To identify entire transposon structures containing the *bla*_OXA-23_ gene in different isolates and determine their location in the target DNA, a cloning procedure was used. Some data had been reported for 6 of 20 isolates (*11*). Total DNA was digested with either *Sac*I or *Sal*I, ligated into the *Sac*I or *Sal*I sites of plasmid pBK-CMV (kanamycin-resistant cloning vector), and the recombinant plasmids were transformed into *Escherichia coli* TOP10, as described (*14*). Recombinant plasmids were selected on trypticase soy agar plates containing amoxicillin (50 mg/L) and kanamycin (30 mg/L). Cloned DNA fragments of several recombinants plasmids were sequenced on both strands by primer walking as described (*11*).

## Results

### Clonal Relatedness of the Isolates

Twenty carbapenem-resistant *A*. *baumannii* isolates were obtained from 15 countries (Table). All isolates were highly resistant to ticarcillin (MIC >256 mg/L) and showed a high level of resistance to ceftazidime (MIC >256 mg/L), except isolates Ab14 (MIC 4 mg/L) 861 and DOS (MIC 8 mg/L). All isolates were resistant to imipenem and meropenem (MIC >16 mg/L) (Table).

**Table:** Characteristics of 20 *bla*_OXA-23_-positive *Acinetobacter baumannii* clinical isolates*

Isolate	Origin	Date of isolation	Specimen	EC	ST†	Copy no. of *bla*_OXA-23_	Genetic location and size, kb	Genetic structure	MIC, μg/mL		
									CAZ	IPM	MEM
240	France	2003 Dec	Tracheal aspirate	II	22/2	1	Chromosome, ≈200‡	Tn*2006*	128	>32	>32
512	Tahiti	2004 Mar	Tracheal aspirate	II	22/2	1	Chromosome, ≈200‡	Tn*2006*	64	>32	>32
761	Vietnam	2005 May	Bile	II	22/2	1	Chromosome, ≈200‡	Tn*2006*	64	>32	>32
810	New Caledonia	2004 Jun	Blood	II	22/2	1	Chromosome, ≈200‡	Tn*2006*	96	>32	>32
863	Thailand	2006 Jun	Urine	II	22/2	1	Chromosome, ≈200‡	Tn*2006*	256	>32	>32
883	Reunion	2006 Jun	Unknown	II	22/2	1	Chromosome, ≈200‡	Tn*2006*	128	>32	>32
Ab13	France	2004 Jun	Urine	II	22/2	2	Chromosome, ≈200,‡ and plasmid, 70	Tn*2006*	128	>32	>32
AUS	Australia	2004 Oct	Urine	II	22/2	1	Chromosome, ≈200‡	Tn*2006*	96	>32	>32
859	South Africa	2006 Jan	Urine	II	22/2	1	Chromosome, ≈200‡	Tn*2006*	128	>32	>32
585	France	2004 Jul	Tracheal aspirate	II	53/2	1	Chromosome, ≈200‡	Tn*2006*	128	>32	>32
614	Libya	2004 Oct	Unknown	I	25/20	1	Plasmid, 130	Tn*2008*	256	>32	16
AS3	UAE†	2006 Oct	Blood	I	25/20	1	Plasmid, 130	IS*Aba1*	256	>32	>32
1190	Bahrain	2008 Mar	Blood	I	25/20	1	Plasmid, 130	IS*Aba1*	256	>32	>32
AS1	UAE	2006 Jul	Blood	I	44/1	1	Chromosome, ≈40‡	Tn*2006*	256	>32	>32
Ab14	Algeria	2004 Dec	Unknown	I	44/1	2	Plasmid, 25, and plasmid, >150	Tn*2007*	4	16	>32
910	Reunion	2006 Oct	Unknown	I	New1/1	1	Plasmid, 130	Tn*2006*	256	16	16
861	Egypt	2005 Nov	Sputum	I	New1/ 1	1	Plasmid, 130	IS*Aba1*	8	32	32
BEL	Belgium	2007 Jul	Respiratory tract	I	New2/ 1	2	Plasmid, 25, and plasmid, >150	Tn*2007*	256	>32	>32
DOS	France	2004 May	Unknown	–	New3/ New	2	Plasmid, 25, and plasmid, >150	Tn*2007*	8	>32	>32
877	Brazil	2006 Jul	Wound	–	New4/15	1	Plasmid, 130	IS*Aba1*	96	>32	>32

*EC, European clone; ST, sequence type; UAE, United Arab Emirates; CAZ, ceftazidime; IPM, imipenem; MEM, meropenem. The MIC for ticarcillin was >256 μg/mL for all 20 isolates. †ST determined by Bartual et al. (*19*) compared with ST determined by Nemec et al. (*20*). ‡Size of chromosome band carrying the *bla*_OXA-23_ gene, as determined by using the I-*Ceu*I technique.

**Figure 2:**
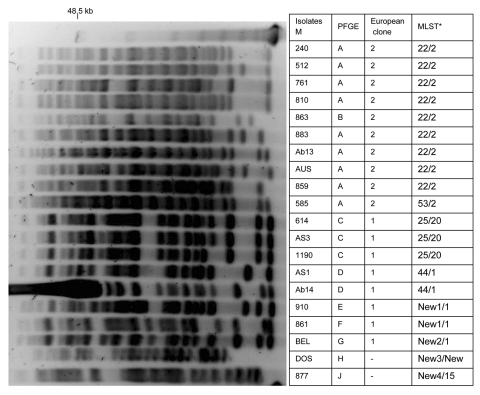
Pulsed-field electrophoresis (PFGE) profiles of *Apa*I-digested genomic DNA from strains of *Acinetobacter baumannii*. PFGE types, European clone types, and multilocus sequence typing (MLST) results are shown. *ST, sequence type determined by Bartual et al. (*19*) compared with ST determined by Nemec et al. (*20*). Lane M, molecular size markers (48.5 kb).

Multiplex PCR for identification of sequence groups showed 10 isolates that belonged to group 1 according to Turton et al. (*18*), eight that belonged to group 2, and 2 isolates that did not belong to groups 1 or 2. The 10 isolates that belonged to group 1 and corresponded to European clone II (*18*) were classified into 2 sequence types (STs), ST22 and ST53, according to MLST analysis (*18*). ST22 (1–3-3–2-2–7-3) was the most frequent type identified. Nine isolates were identified: 2 from France and 1 each from Vietnam, New Caledonia, Thailand, Australia, Tahiti, Reunion, and South Africa. A single European clone II isolate was classified as ST53 (1–3-3–2-2,3-3), a single-locus variant of ST22. Among 10 other isolates, 8 belonged to group 2 (corresponding to European clone I). Four STs were identified: ST25 (10–12–4–11–1–9–5) (Libya, United Arab Emirates, and Bahrain), ST44 (10–12–4–11–4–9–5) (United Arab Emirates and Algeria), and 2 new STs, 1 for isolates from Reunion and Egypt (10–12–4–11–4–16–5) and another related ST identified in the single isolate from Belgium (10–12–4–11–4,4–5). These 4 STs differ by 1 locus. The 2 most recent isolates from France and Brazil did not belong to European clones I or II and corresponded to 2 STs (1–22–3-11–1-9–7 and 12–18–12–1-15–9-19, respectively) (Table). Although 8 STs were identified in this collection, 9 pulsotypes were characterized by PFGE according to the criteria of Tenover et al. (23) (Figure 2).

According to MLST analysis developed by Nemec et al. (*20*), all isolates that belonged to European clone II had the same sequence type (ST2) (2,2-2,2-2,2-2), including isolate 585, which had a distinct but related ST in the first analysis. Among isolates that belonged to European clone I, two sequence types were determined: ST20 (3–1-1,1-5–1-1) (Libya, United Arab Emirates, Bahrain) and ST1 (1,1-1,1-5–1-1) (United Arab Emirates, Reunion, Egypt, Belgium, Algeria). Isolates 910 (Reunion), 861 (Egypt), and BEL (Belgium) were included in ST1. These isolates had a distinct ST according to methods of Bartual et al. (*19*). The 2 most recent isolates were classified into 2 STs, a new ST (3–2-2,2-5–4-8) for isolate DOS (France) and ST15 (6,6-8–2-3–5-4) for isolate 877 (Brazil) (Table).

### Location and Transferability of the *bla*_OXA-23_ Gene

Location of the *bla*_OXA-23_ gene was evaluated by using the I-*Ceu*I method. Eleven isolates had the *bla*_OXA-23_ gene on the chromosome, with a hybridization signal for an ≈40-kb band for isolate AS1 and an ≈200-kb band for 10 isolates (Table). Nine isolates carried the *bla*_OXA-23_ gene on a plasmid and 1 isolate had 2 copies of the *bla*_OXA-23_ gene, 1 on the chromosome and 1 on a 7–kb plasmid (Table).

To examine the copy number of the *bla*_OXA-23_ gene in different *A*. *baumannii* genomes, we performed Southern blot hybridization on *Eco*RI-digested DNA fragments using a 589-bp DNA probe specific for the *bla*_OXA-23_ gene. Sixteen isolates showed only 1 copy of the *bla*_OXA-23_ gene. Isolates BEL, Ab14, and DOS had 2 copies of the *bla*_OXA-23_ gene on different plasmids, and Ab13 had 1 copy on the chromosome and 1 copy on a plasmid according to results of the I-*Ceu1* technique.

Mating-out assays were performed by using the 10 plasmid-positive strains as donor strains and rifampin-resistant *A*. *baumannii* BM4547 as the recipient strain. Five transconjugants were obtained; all had a 130-kb plasmid that did not provide additional antimicrobial drug resistance to the *A*. *baumannii* recipient strain, except in 1 case (co-resistance to kanamycin and amikacin on a *bla*_OXA-23_–carrying plasmid that originated from isolate 1190). Plasmids carrying the *bla*_OXA-23_ gene in isolates Ab14, DOS, BEL, and 877 were not self-transferable (Table) (*24*).

### Variability of Genetic Structures Flanking the *bla*_OXA-23_ Gene

The 10 isolates that belonged to European clone II had a *bla*_OXA-23_ gene that was part of Tn*2006*. The 9-bp direct repeat (DR) that corresponded to duplication of the Tn*2006* target site, which was consistent with a transposition event, was identified in the 9 ST22/ST2 isolates. Tn*2006* was inserted in different locations on the chromosomes of those isolates (Table). For isolates 240, 512, 810, 859, 883, and Aus, the insertion occurred between 2 genes encoding hypothetical proteins (DR: GTCATTTAA) (Figure 1). In isolate 761, transposon Tn*2006* was located between a gene encoding a hypothetical protein and a gene encoding an isoleucyl tRNA synthase (DR: ATTCGCGGG). In isolate 863, Tn*2006* was identified between a gene encoding a cytochrome D terminale oxidase and a putative transposase (DR: ATAATTATT). In isolate 585, Tn*2006* was located between a gene encoding a hypothetical protein and a *sul1* gene (DR: ATTCGCGGG). The plasmid-borne *bla*_OXA-23_ gene identified in isolate Ab13 was also part of Tn*2006* but was inserted into the *sul* gene that encoded a putative sulfonamide resistance determinant (DR: ATTCGCGGG).

Isolates that belonged to European clone I had diverse genetic structures at the origin of *bla*_OXA-23_ acquisition. Two isolates had transposon Tn*2006*: one on the chromosome (AS1) and 1 on a plasmid (910). Transposon Tn*2007* was identified in 3 isolates; it was specific for the same open reading frame in 2 isolates (BEL and Ab14) (Figure 2). Only 1 copy of IS*Aba1* was identified upstream of the *bla*_OXA-23_ gene in isolates AS3, 1190, 861, and 877. Transposon Tn*2008* was identified only in isolate 614 (Figure 1). Sequences of these specific genetic structures have been deposited in Genbank (accession nos. EF127491, EF059914, GQ861438, and GQ861439).

## Discussion

This study was conducted to define which features may explain the worldwide dissemination of the *bla*_OXA-23_ gene in *A*. *baumannii*. Isolates were from the Middle East, Europe, and Asia; there were no isolates from North America. Except for 2 isolates, the isolates investigated in this study belonged to European clones I or II. Clustering of *A*. *baumannii* isolates was determined by MLST and PFGE; our collection was composed of 13 PFGE types corresponding to 9 STs. Eight STs were identified among the OXA-23–producing *A*. *baumannii*; the most common STs were ST22/ST2 found in France (n = 2), Vietnam, New Caledonia, Thailand, Australia, Reunion, South Africa, and Tahiti. Spread of *bla*_OXA-23_–positive *A*. *baumannii* isolates that belong to clone ST22 has been demonstrated in South Korea (*25*). Analysis of the target site of *bla*_OXA-23_ acquisition showed that in the same clone, such as ST22, acquisition of the Tn*2006* composite transposon had occurred at different positions in the *A*. *baumannii* genome, which suggested that Tn*2006*-mediated acquisition of *bla*_OXA-23_ may occur as independent events, or that Tn*2006* is a structure that is mobile in a given genome. A single clone could have different genetic structures at the origin of the *bla*_OXA-23_ acquisition.

We showed that the *bla*_OXA-23_ gene associated with Tn*2006* could be located on the chromosome or a plasmid. This result agrees with our recent findings, which showed that Tn*2006* is capable of transposition (*13*). We have also observed that 5 isolates with different sequence types (STNew1, ST25) harbored a similar 130-kb plasmid. The same strains with the same genetic structure were identified in 8 countries in different parts of the world.

In conclusion, the current worldwide dissemination of the *bla*_OXA-23_ gene is driven by >7 MLST types associated with different genetic structures and plasmids. We have identified complex and dynamic spreading of *bla*_OXA-23_ that will be difficult to control because this spread is not associated with a single entity.
